# Lifestyle and Symptom Risk Factors for Dry Eye Disease in Asian Gout Population: A Population-Based Case–Control Study

**DOI:** 10.3390/jcm11247378

**Published:** 2022-12-12

**Authors:** Chia-Yi Lee, Ie-Bin Lian, Yan-Ni Jhan, Shun-Fa Yang, Chao-Kai Chang

**Affiliations:** 1 Institute of Medicine, Chung Shan Medical University, Taichung 40201, Taiwan; 2 Nobel Eye Institute, Taipei 100008, Taiwan; 3 Department of Ophthalmology, Jen-Ai Hospital Dali Branch, Taichung 41265, Taiwan; 4 Institute of Statistical and Information Science, National Changhua University of Education, Changhua 50007, Taiwan; 5 Department of Medical Research, Chung Shan Medical University Hospital, Taichung 40201, Taiwan; 6 Department of Optometry, Da-Yeh University, Chunghua 51500, Taiwan

**Keywords:** dry eye disease, gout, visual display terminal, alcohol, lifestyle

## Abstract

We aim to investigate the lifestyle and symptom factors related to dry eye disease (DED) presence in patients with persistent gout using the Taiwan Biobank (TWB) database. A retrospective case–control study was conducted, and patients with a history of gout longer than 10 years were enrolled in the persistent-gout group. Each persistent-gout patient was age- and sex-matched to two non-gout individuals who served as the control group, and we included a total of 973 and 1946 patients in the persistent-gout group and non-gout groups. The main outcome of our study is the presence of DED and the distribution of several lifestyle and symptom risk factors of DED in persistent-gout and non-gout individuals. Logistic regression considering the age and sex and interaction test was applied to estimate the correlation of each lifestyle and symptom risk factor to DED in the two groups. A total of 95 and 140 DED events were found in the persistent-gout and non-gout groups, with a significantly higher percentage in the persistent-gout population (aOR: 1.167, 95% CI: 1.073–3.855, *p* = 0.0415). The visual display terminal (VDT) user (*p* = 0.0026) and persistent alcohol drinking (*p* = 0.0384) were associated with DED more often in the persistent-gout population than the non-gout population. Moreover, the percentages of DED in the patients with gout intervals of 10–20 years and more than 20 years were statistically insignificant (aOR: 1.042, 95% CI: 0.886–1.910, *p* = 0.5279). In conclusion, VDT usage and persistent alcohol drinking are prominent lifestyle and symptom risk factors for DED occurrence in patients who have experienced gout for more than 10 years.

## 1. Introduction

Dry eye disease (DED) is a common ocular disease, and a great prevalence of DED has been observed in the Asian population [1]. Advanced DED can develop in approximately 20% of the general DED population [2]. Regarding the therapeutic options of DED, some methods, such as warm compressing, artificial tears, overnight ophthalmic ointment, and intense pulsed light therapy, have been applied, with satisfactory outcomes [3,4,5]. When it comes to the risk factors of DED, systemic diseases, including keratoconjunctivitis sicca, rheumatoid arthritis, and systemic lupus erythematous, correlate to DED episodes [2]. Additionally, certain medications, such as cyclophosphamide and methotrexate, are associated with the presence of DED [2].

The presence of DED was shown to have a positive relationship with pre-existing lifestyle factors in a previous study: prolonged digital screen exposure contributes to a higher rate of DED incidence [6]. Additionally, patients using visual display terminal (VDT) devices during daily tasks had a significantly higher incidence of DED [7]. Other lifestyle and symptom risk factors for DED include smoking at any time and insufficient sleeping hours in daily life [8,9], while a high amount of exercise can decrease the rate of DED [10]. Nevertheless, whether the lifestyle and symptom risk factors contribute to equal effects in the general population and population with specific disorders has not been fully elucidated.

Gout is a hyperuricemia-related inflammatory disorder with crystal deposition that mainly damages the joints and tissues [11,12,13]. Regarding the relationship between gout and DED, several previous researchers have surveyed this issue with conflicting results [14,15,16,17]. On the other hand, studies evaluating the potential lifestyle and symptom risk factors for DED in the gout population compared to the general population are rare. Both gout and DED are diseases with an inflammatory nature, and a prolonged gout course was correlated to a higher incidence of DED in previous literature [17]. Thus, we speculated that various non-disease factors, including certain lifestyle and symptom factors, would relate to DED presence in patients with persistent gout who experience prolonged inflammation compared to the general population.

Consequently, we aim to investigate the potential lifestyle and symptom factors for DED presence in patients with gout for more than 10 years. We used the Taiwan Biobank (TWB) database as a resource, and the influence of age and sex were considered in our analytic models.

## 2. Materials and Methods

### 2.1. Data Source

The TWB database was set up by the Academia Sinica of Taiwan, and more than 30 institutions recruited participants for TWB. These institutions are spread out throughout the northern, central, southern, and eastern areas of Taiwan. In 2012–2022, there were 172,078 participants entered the TWB project, and a venous blood sample and questionnaire of each patient were collected after signing the informed consent produced by the TWB. The data available in the questionnaire of TWB include age, sex, residence area, socioeconomic status, occupation, lifestyle habit, specific and severe symptoms, personal history, and medical history. Moreover, basic body information, biochemistry profiles, and genetic analyses are also available in the TWB database if a venous blood sample analysis was completed.

### 2.2. Subject Selection

**Figure 1:**
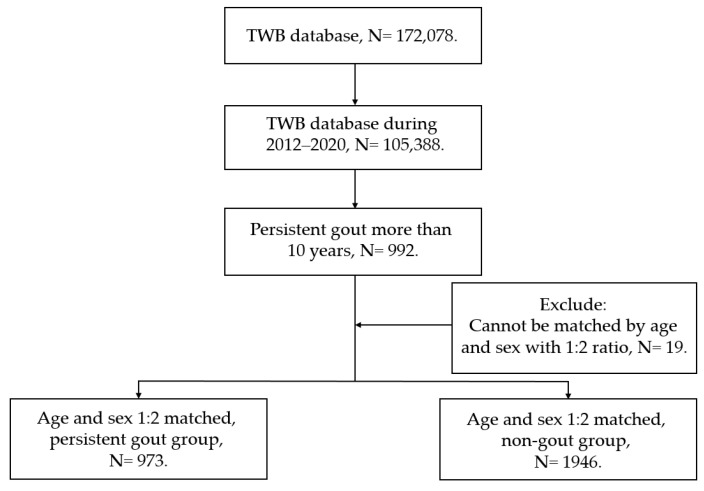
The flowchart of selection process in the current study. TWB: Taiwan Biobank. N: number.

Participants were defined as having persistent gout if their documents in the TWB database revealed (1) a diagnosis of gout according to the questionnaire filled by each patient, (2) the duration of gout was more than 10 years according to the same questionnaire, (3) the year of gout diagnosis was available in the TWB database, (4) they were of Han ethnicity, and (5) the patient participated in the research program in 2012–2020. Moreover, each individual in the persistent-gout group was age- and sex-matched to two patients without any gout diagnosis with Han ethnicity and recruited in 2012–2020, which constituted the control group. We matched the patients via age and sex since both are known risk factors for DED [2]. After the whole procedure, 973 and 1946 individuals were included in the persistent-gout group and non-gout group, respectively. The residence area, marriage status, and education level of the two groups were obtained from the same questionnaire in the TWB. The purpose of including these demographic data was to complete the survey of demography and increase the integrity of our study. The patients who did not fill out the questionnaire items were not included in the current research. The flowchart of our patient selection process is presented in Figure 1.

### 2.3. Main Outcome and Confounders

The main outcome of our study is the presence of DED, which needs to include the subsequent criteria: (1) the DED diagnosis in the questionnaire was completed by the patient; (2) the year of DED diagnosis is available. The questions for establishing DED existence include the presence of dryness. In addition to the main outcome, we obtained multiple lifestyles and symptom factors based on the questionnaire, which included VDT use, chronic pain, persistent cigarette smoking, persistent alcohol drinking, sedentary lifestyle, persistent betelnut chewing, vegan diet, oily and salty diet habit, and family history of gout. The existence of chronic pain was defined as pain at any location which persisted for more than three months in the questionnaire. For details of other factors, the VDT user was patients who had ever worked in the retail business, wholesale industry, international trade, financial industry, communication industry, real estate industry, insurance business, law practice, public administration, investment industry, education industry, medical practice, mass communication, cultural industry, and international institutions. The reason for the VDT inclusion criteria is that these occupations frequently involve employees using their cell phones or computers to complete their daily work. We defined persistent alcohol drinking, betelnut chewing, and cigarette smoking as the current usage of these substances at the time of the questionnaire’s completion, where the substance usage has persisted for more than five years. We regarded a sedentary lifestyle as the lack of an exercise habit ever, and an oily and salty diet habit was based on positive answers concerning a high-fat and high-salt diet in more than 4 of 8 questions about diet habits. We defined a participant as vegan if a vegan diet status was confirmed at the time of the questionnaire and had persisted for at least five years. Finally, the family history of gout was counted if any of the participant’s relatives had been diagnosed with gout.

### 2.4. Statistical Analysis

SAS version 9.4 (SAS Institute Inc, Cary, NC, USA) was utilized for all the analyses in our study. The basic information and demographic data of the two groups were presented by descriptive analyses. We applied the Shapiro–Wilk test to test the normality, and the age distributions of the two groups had a normal distribution (both *p* = 0.253 due to matching). We used an independent T-test to compare the age at recruitment in the persistent-gout and non-gout groups, and the Chi-square test was applied to analyze the rest of the demographic data in the two populations. The incidences of DED in gout and non-gout groups were analyzed via logistic regression, which incorporated the effect of age and sex and yielded the adjusted odds ratio (aOR), 95% confidence interval, and *p* value. We then separated the persistent-gout group into patients with DED and patients without DED, and the Chi-square test was exploited to investigate the distribution of different lifestyle and symptom factors in the persistent-gout-with-DED and persistent-gout-without-DED subgroups. Similarly, the non-gout group was divided into those with DED and those without DED, and the distributions of lifestyle and symptom risk factors in the two subgroups were analyzed via the Chi-square test again. In the next step, we used an interaction test to evaluate whether the correlation between specific lifestyle and symptom factors and DED presence is higher in the persistent-gout group than in the non-gout group. In addition, we divided the persistent-gout population into those with a gout interval from 10 to 20 years and individuals with a gout interval of more than 20 years, and logistic regression was used to produce the aOR with the correlating 95% CI and *p* value. A *p* value lower than 0.05 is seen as statistically significant in our study, and a *p* value lower than 0.0001 is demonstrated as *p* < 0.0001.

## 3. Results

The basic information of the two groups is shown in Table 1. The mean age and percentage of males were 53.22% and 93.15% in both groups, respectively, after the matching process. The persistent-gout group showed a lower educational level (*p* = 0.0037) than the non-gout group. Nevertheless, other demographic data, including the marriage status and place of residence, did not show a significant difference between the persistent-gout and non-gout groups (all *p* > 0.05) (Table 1).

In the whole study population, 95 and 140 episodes of DED were recorded in the persistent-gout and non-gout groups, respectively. The DED events in the persistent-gout group were significantly higher than that in the non-gout group (aOR: 1.167, 95% CI: 1.073–3.855, *p* = 0.0415) (Table 2). In a subgroup analysis of the persistent-gout population, the persistent-gout individuals with DED showed a significantly higher mean age (*p* < 0.0001), a lower proportion of male sex (*p* < 0.0001), a higher ratio of VDT use (*p* < 0.0001), a higher ratio of chronic pain (*p* = 0.0101), a higher rate of persistent alcohol drinking (*p* = 0.0001), and a higher rate of persistent cigarette smoking (*p* = 0.0123) compared to the persistent-gout patients without DED (Table 3). Concerning the non-gout population, the patients with DED illustrated an older mean age (*p* < 0.0001), a lower proportion of male sex (*p* < 0.0001), a higher rate of VDT use (*p* = 0.0013), a higher rate of chronic pain (*p* = 0.0068), and a higher ratio of persistent cigarette smoking (*p* = 0.0476) (Table 3). When analyzing the correlation of lifestyle and symptom factors to DED in the two groups, the usage of VDT (*p* = 0.0026) and persistent alcohol drinking (*p* = 0.0384) were correlated to DED presence more frequently in the persistent-gout group compared to the non-gout group (Table 3).

In the subgroup analysis of persistent-gout patients, 43 episodes of DED were recorded in patients with a gout duration of 10–20 years, and 52 DED events were presented in individuals with a gout duration of more than 20 years. The logistic regression demonstrated a non-significant difference in the DED ratio between the two gout-subgroups (aOR: 1.042, 95% CI: 0.886–1.910, *p* = 0.5279) (Table 4).

## 4. Discussion

In this study, the percentage of DED was significantly higher in the persistent-gout population with a disease duration of more than 10 years compared to the non-gout population. Additionally, the persistent-gout patients with VDT application and persistent alcohol drinking presented with DED more frequently compared to the non-gout population with VDT usage and persistent alcohol drinking. On the other hand, the gout patients with a disease duration longer than 20 years did not have a higher DED presence compared to those diagnosed with gout for 10–20 years.

DED is an ocular disease with an inflammatory process and a tear film insufficiency [4,18,19]. The tear film in DED patients contains a high amount of inflammatory markers and cytokines [2]. In a previous study, the interleukin family showed a significantly higher value than the normal population. Another research revealed a 100-fold higher concentration of matrix metalloproteinase-9 in the tear film of DED patients [20]. On the other hand, ocular surface damage is related to the formation of DED [4]. Damage to the ocular surface enhances immune-cell and releases inflammatory cytokines [21], and these mediators can lead to higher tear film hyperosmolarity and nuclear factor κB signaling pathways, which elevate inflammation levels [22]. Consequently, these inflammation pathways lead to subsequent DED. Thus, this situation repeats and the severity of DED and ocular surface damage increase progressively [2]. Moreover, certain systemic inflammatory diseases can elevate the probability of DED via the inflammatory pathway. Sjogren syndrome is a prominent systemic inflammatory disease that correlates to the occurrence of DED [1]. The presence of ocular dryness was found in 95% of individuals with Sjogren syndrome [23], and the objective DED severity in patients with Sjogren syndrome was significantly higher than that in the general population with DED [24]. In addition to Sjogren syndrome, rheumatic arthritis is correlated to DED, and DED severity is associated with the disease duration of rheumatic arthritis [24]. Additionally, systemic lupus erythematosus is associated with DED with reduced tear film production and stability [25]. Gout is also an inflammatory disorder in which both the interleukin and nuclear factor κB signaling pathways are involved in disease formation [12,13]. Moreover, a higher level of aggregated neutrophils and tumor necrosis factor α were observed in the tophus of gout patients [26,27]. Because of the similar inflammatory process in gout and DED [22,27], we proposed that DED may be associated with patients with persistent gout, and certain lifestyle and symptom risk factors may be more associated with DED in such a population than the general population. This concept was partially supported by the results of the current research.

The current study showed a significant correlation between DED and persistent gout for more than 10 years. In some previous studies, gout was associated with subsequent DED [16,17]. On the contrary, other previous studies illustrated an insignificant relationship between DED and gout [14]. The possible reason for this conflict is that we only selected patients diagnosed with gout for more than 10 years in our study compared to the general gout population in previous publications [14]. The longer duration of gout puts the human body under greater inflammatory stress, and a higher DED ratio may thus reveal itself. The above evidence implies that gout may be associated with DED, but the correlation is not universally strong and may relate to the disease duration of gout. However, patients with gout for 10–20 years showed a similar ratio of DED compared to those with gout disease for more than 20 years. This finding may imply that 10 years is a threshold for a higher rate of DED in the gout population. On the other hand, the persistent-gout patients with DED showed a higher percentage of VDT use and persistent alcohol drinking than the general DED population with these lifestyle and symptom risk factors. To our knowledge, research has scarcely reported such a relationship before. Persistent VDT use is a major risk factor for DED presence in previous publications [7]. The excessive exposure to air and tear film evaporation in VDT use is the possible mechanism for DED presence in such a population [7]. Additionally, the ocular surface may be more vulnerable to this condition under elevated inflammation in persistent gout. In addition, persistent alcohol drinking is related to DED occurrence in patients with persistent gout, while alcohol drinking is not a known risk factor for DED. Although the development of gout is associated with alcohol drinking [11], we analyzed the effect of gout and alcohol drinking separately in different statistical models. We found that persistent gout is associated with DED, and the correlation between alcohol and DED only exists in the presence of persistent gout but not in the general population. We speculated that since alcohol drinking is a risk factor for gout and the associated inflammatory response [11], persistent alcohol drinking and repeated gout exacerbation during persistent gout relate to the presence of DED. Still, further study is needed to prove this speculation.

When evaluating the lifestyle risk factors for DED in the persistent-gout population and non-gout population, old age, female sex, VDT use, chronic pain, and persistent cigarette smoking correlate to a higher percentage of DED events in both groups. Old age and female sex are both well-known risk factors for DED occurrence [2]. The prominent effect of chronic pain in the pathophysiology of DED has been established in previous articles [28,29], as patients with gout experience pain and swelling around the joint area, which can cause chronic pain [13]. VDT use is an established risk factor for DED in the general population [5,6,7]. Thus, it could relate to a higher percentage of DED in patients with persistent gout and without gout. Persistent cigarette smoking has been proven to be associated with DED presence [30,31]. Thus, it is reasonable that persistent cigarette smoking significantly correlates to DED presence in both groups. In terms of the insignificant relationship between persistent alcohol drinking and DED in the non-gout population, a possible explanation is that the inflammatory response from solitary persistent alcohol drinking is not strong enough to trigger the DED cycle in the general population. Concerning the basic characteristics and the percentage of lifestyle and symptom risk factors in the persistent-gout and non-gout groups, the ratio of low educational level was significantly higher in the persistent-gout population. Moreover, several negative lifestyle and symptom factors, such as persistent alcohol drinking, persistent cigarette smoking, and persistent betelnut chewing, were higher in the persistent-gout population. This may indicate a poorer health condition in patients with persistent gout.

In terms of epidemiology, DED affects about 6.8% of patients in the United States and 9.1% of individuals in the Netherlands [3,8]. Moreover, gout is the most prevalent inflammatory arthritis, with a prevalence rate of about 0.79% in the male population [12]. In the above two diseases, VDT use is more prevalent. According to a review article, VDT users that use the internet reached 63% at the end of 2020 [7]. Alcohol is a substance that has been abused for centuries, and more than 60% of Americans consume alcohol daily [32]. Since the above disorders occur commonly and influence numerous individuals, any relationship among them could be illustrated to recognize potential victims of DED.

There are some limitations in this study. First, the case–control design cannot show a causal relationship between lifestyle and symptom factors and the presence of DED. In addition, all the diagnoses in the current study were only based on the description of participants in their filled-out questionnaires. Thus, some critical information, such as the disease duration, consistency of patients’ DED diagnosis, the severity of DED, examination results, treatment effect, prognosis, and recurrence of both gout and DED, cannot be accessed. Additionally, we may have lost some silent DED cases since we used a questionnaire survey rather than a real ophthalmic assessment as the criteria for DED. Moreover, some systemic diseases and medications can contribute to DED events [2], but we cannot use these data in the analysis model in this study because the questionnaire items did not include those factors. Moreover, we excluded many patients because they did not answer certain questions or did not have a gout interval that had lasted for more than 10 years. Thus, more than half of the participants with a gout history were excluded. However, the total number of participants in the current study is 2919, which is not inferior to previous literature evaluating DED [9,10,33]. Accordingly, the statistical power of our study is adequate.

## 5. Conclusions

In conclusion, VDT use and persistent alcohol drinking are associated with DED presence more often in persistent-gout patients than non-gout individuals. Furthermore, a gout duration of more than 10 years could be a threshold for a higher rate of DED presence. Consequently, a periodical external eye exam may be recommended for gout patients with a disease interval of longer than 10 years to diagnose potential DED. Additionally, a decrease in VDT use and alcohol drinking should be suggested in this persistent-gout population, whether diagnosed with DED or not, to prevent further DED episodes. A further large-scale prospective study to investigate the influence of these lifestyle and symptom factors on the therapeutic effectiveness of DED in patients with persistent gout is mandatory.

## Figures and Tables

**Table 1:** Characteristics of the study population.

Variable	Persistent Gout (N = 973)	Non-Gout (N = 1946)	*p* Value
Age	53.22	53.22	1.000
Sex (male)	902 (93.15%)	1803 (93.15%)	1.000
Education			0.0037 *
Elementary school	55 (5.48%)	79 (4.10%)	
High school	352 (36.21%)	675 (34.68%)	
University and above	566 (58.31%)	1192 (61.22%)	
Marriage			0.0561
Unmarried	78 (7.89%)	189 (9.63%)	
Married	807 (83.30%)	1596 (82.29%)	
Divorced	80 (8.13%)	133 (6.77%)	
Widowed	8 (0.68%)	28 (1.31%)	
Place of residence			0.2911
Northern	308 (31.61%)	649 (33.34%)	
Central	274 (28.17%)	510 (26.18%)	
Southern	339 (34.92%)	658 (33.81%)	
Eastern	49 (4.96%)	121 (6.60%)	
Outlying Islands	3 (0.34%)	1 (0.07%)	

N: number. * Denotes significant difference between the two groups.

**Table 2:** Incidence of dry eye in the persistent-gout and non-gout groups.

Group	DED (%)	Non-DED (%)	Total	aOR (95% CI)	*p* Value
Persistent gout	95 (9.76)	878 (90.24)	973		
Non-gout	140 (7.21)	1806 (92.79)	1946		
Total	235	2684	2919	1.167 (1.073–3.855)	0.0415 *

DED: dry eye disease, aOR: adjusted odds ratio, CI: confidence interval. * Denotes significant difference between the two groups.

**Table 3:** The lifestyle and symptom factor and dry eye presence in the persistent-gout and non-gout populations.

Variable	Persistent Gout Group (N = 973)			Non-Gout Group (N = 1946)			Interaction *p*
	DED (N = 95)	Non-DED (N = 878)	*p*1 Value	DED (N = 140)	Non-DED (N = 1806)	*p*2 Value	
Age (mean ± SD)	58.24 ± 0.39	52.77 ± 0.45	<0.0001 *	57.45 ± 0.60	52.96 ± 0.42	<0.0001 *	0.6336
Sex (male)	72 (75.87%)	840 (95.72%)	<0.0001 *	107 (76.51%)	1669 (92.43%)	<0.0001 *	0.7528
Family history of gout	62 (65.16%)	564 (64.23%)	0.9952	89 (63.57%)	1167 (64.64%)	0.9456	0.9012
VDT user	83 (87.38%)	506 (57.63%)	<0.0001 *	97 (69.28%)	1003 (55.54%)	0.0013 *	0.0026 ^†^
Chronic pain	12 (12.49%)	88 (10.03%)	0.0101 *	18 (12.86%)	185 (10.25%)	0.0068 *	0.5502
Persistent alcohol drinking	23 (24.20%)	156 (17.76%)	0.0001 *	29 (20.71%)	351 (19.43%)	0.0731	0.0384 ^†^
Persistent cigarette smoking	15 (15.69%)	114 (13.01%)	0.0123 *	19 (13.57%)	237 (12.12%)	0.0476 *	0.7424
Persistent betelnut chewing	49 (51.28%)	465 (52.95%)	0.6372	71 (50.71%)	908 (50.29%)	0.9584	0.8311
Sedentary lifestyle	5 (5.26%)	38 (4.32%)	0.1475	8 (5.71%)	90 (5.00%)	0.7069	0.1935
Oily and salty diet habit	1 (1.05%)	8 (0.91%)	0.8763	3 (2.14%)	36 (1.99%)	0.6413	0.8880
Vegan	11 (11.58%)	87 (9.89%)	0.1905	16 (11.42%)	212 (11.74%)	0.8598	0.0946

DED: dry eye disease, N: number, SD: standard deviation, VDT: visual display terminal, * Denotes significant difference between the dry eye disease and non-dry-eye disease subgroups. Interaction *p*: comparison of *p* value for each variable between the persistent-gout group and non-gout group; ^†^ denotes significant difference between the two groups.

**Table 4:** The distribution of dry eye in persistent-gout patients with different disease periods.

Period	DED (%)	Non-DED (%)	Total	aOR (95% CI)	*p* Value
Gout for 10–20 years	43 (9.15)	427 (90.85)	470		
Gout for >20 years	52 (10.34)	451 (89.66)	503		
Total	95	878	973	1.042 (0.886–1.910)	0.5279

DED: dry eye disease, aOR: adjusted odds ratio, CI: confidence interval.

## Data Availability

The data used in this study are available upon reasonable request to the corresponding author.
